# Data-driven approach to integrating genomic and behavioral preclinical traumatic brain injury research

**DOI:** 10.3389/fbioe.2022.887898

**Published:** 2023-01-10

**Authors:** J. Russell Huie, Jessica L. Nielson, Jorden Wolfsbane, Clark R. Andersen, Heidi M. Spratt, Douglas S. DeWitt, Adam R. Ferguson, Bridget E. Hawkins

**Affiliations:** ^1^ Weill Institutes for Neurosciences, Brain and Spinal Injury Center, Department of Neurosurgery, University of California, San Francisco, San Francisco, CA, United States; ^2^ San Francisco Veterans Administration Medical Center, San Francisco, CA, United States; ^3^ Department of Psychiatry and Behavioral Sciences, Institute for Health Informatics, University of Minnesota, Minneapolis, MN, United States; ^4^ The Moody Project for Translational Traumatic Brain Injury Research, Department of Anesthesiology, University of Texas Medical Branch, Galveston, TX, United States; ^5^ Office of Biostatistics, Department of Preventive Medicine Population Health, University of Texas Medical Branch, Galveston, TX, United States; ^6^ Research Innovation and Scientific Excellence (RISE) Center, School of Nursing, University of Texas Medical Branch, Galveston, TX, United States; ^7^ Biostatistics Department, UT MD Anderson, Houston, TX, United States

**Keywords:** TBI, traumatic brain injury, data-driven learning, behavior analysis, multivariate statistical analyses, genomic

## Abstract

Understanding recovery from TBI is complex, involving multiple systems and modalities. The current study applied modern data science tools to manage this complexity and harmonize large-scale data to understand relationships between gene expression and behavioral outcomes in a preclinical model of chronic TBI (cTBI). Data collected by the Moody Project for Translational TBI Research included rats with no injury (naïve animals with similar amounts of anesthetic exposure to TBI and sham-injured animals), sham injury, or lateral fluid percussion TBI, followed by recovery periods up to 12 months. Behavioral measures included locomotor coordination (beam balance neuroscore) and memory and cognition assessments (Morris water maze: MWM) at multiple timepoints. Gene arrays were performed using hippocampal and cortical samples to probe 45,610 genes. To reduce the high dimensionality of molecular and behavioral domains and uncover gene–behavior associations, we performed non-linear principal components analyses (NL-PCA), which de-noised the data. Genomic NL-PCA unveiled three interpretable eigengene components (PC2, PC3, and PC4). Ingenuity pathway analysis (IPA) identified the PCs as an integrated stress response (PC2; EIF2-mTOR, corticotropin signaling, etc.), inflammatory factor translation (PC3; PI3K-p70S6K signaling), and neurite growth inhibition (PC4; Rho pathways). Behavioral PCA revealed three principal components reflecting the contribution of MWM overall speed and distance, neuroscore/beam walk, and MWM platform measures. Integrating the genomic and behavioral domains, we then performed a ‘meta-PCA’ on individual PC scores for each rat from genomic and behavioral PCAs. This meta-PCA uncovered three unique multimodal PCs, characterized by robust associations between inflammatory/stress response and neuroscore/beam walk performance (meta-PC1), stress response and MWM performance (meta-PC2), and stress response and neuroscore/beam walk performance (meta-PC3). Multivariate analysis of variance (MANOVA) on genomic–behavioral meta-PC scores tested separately on cortex and hippocampal samples revealed the main effects of TBI and recovery time. These findings are a proof of concept for the integration of disparate data domains for translational knowledge discovery, harnessing the full syndromic space of TBI.

## Introduction

Traumatic brain injury (TBI) is complex and heterogenous in nature and can produce long-lasting effects clinically that can be replicated with preclinical models (Masel and DeWitt, 2010). To date, there are no FDA-approved therapies specifically for the treatment of TBI. This may be in part due to limitations in preclinical study design and data analysis (DeWitt et al., 2018). Multi-site groups are efficient at evaluating treatments for TBI (CENTER-TBI and TRACK-TBI for clinical studies) and preclinically through efforts such as The Moody Project for Translational TBI Research (Moody Project) and Operation Brain Trauma Therapy (OBTT (Kochanek et al., 2011; Yue et al., 2013; Maas et al., 2015; DeWitt et al., 2018).

The neurotrauma field is facing a big data challenge, and the large collaborative efforts have been at the forefront in designing protocols for dealing with variable datasets (Huie et al., 2018; Hawkins et al., 2020). The Moody Project has accumulated large amounts of highly varied datasets including gene expression, proteomics, histology, behavior, and surgical data over multiple timepoints and with more than one brain region and injury status group.

The main purpose of this effort is to create an example repository of completed studies in preclinical TBI and develop syndromic analytical workflows that 1) capture the complexity of the study design and 2) render outcome testing through multidimensional machine learning approaches to increase the effect size resolution for hypothesis testing of treatment trials. Prior applications of these methodologies to preclinical TBI and clinical TBI have demonstrated the utility of multiscalar ‘omics (syndromics) for TBI and spinal cord injury (SCI) (Ferguson et al., 2011; Haefeli et al., 2017; Nielson et al., 2017). The current study aims to expand upon these efforts, capturing the full spectrum of bio-behavioral and molecular changes that result from TBI, and help resolve treatment approaches that reproduce results across diverse endpoints, increasing potential for translation into clinical trials in humans.

## Materials and methods

### Animals

This study was conducted in a facility approved by the American Association for the Accreditation of Laboratory Animal Care (AAALAC), and all experiments were performed in accordance with the National Institutes of Health Guide for the Care and Use of Laboratory Animals (eighth edition, National Research Council) and approved by the Institutional Animal Care and Use Committee of the University of Texas Medical Branch (UTMB). Adult, male, Sprague–Dawley rats (Charles Rivers Laboratories, Inc., Wilmington, MA), weighing 350–400 g, were group-housed (two rats of similar injury status per cage) and had access to food and water *ad libitum* in a vivarium with the following constant conditions: light cycle (600–1800), temperature (21°C–23°C), and humidity (40%–50%). Unless noted, all animals were provided with enrichment materials, such as a cardboard tube, in their home cage.

### Fluid percussion injury

Animals were anesthetized with 4% isoflurane in an anesthetic chamber, intubated, and mechanically ventilated with 1.5–2.0% isoflurane in O_2_:room air (70:30) using a volume ventilator (EDCO Scientific, Chapel Hill, NC). Rats were prepared for parasagittal fluid-percussion TBI as previously described (Sell et al., 2017). Briefly, animals were placed in a stereotaxic head frame, and the scalp was sagittally incised. A 4.0-mm-diameter hole was trephined 2.0 mm into the skull to the right of the sagittal suture and midway between lambda and bregma. Then, a modified 20-gauge Luer Lok syringe hub (Becton-Dickinson, Franklin Lakes, NJ) was placed over the exposed dura, bonded in place with cyanoacrylic adhesive, and covered with dental acrylic. Animals with punctured dura were excluded from the study. Isoflurane was temporarily discontinued, and rats were connected to the fluid percussion trauma device (Custom Design and Fabrication, Virginia Commonwealth University, VA) using a long tube (high-pressure tubing length 41 cm, volume 2 ml, Baxter #2C5643) connected at one end to the FPI device and the other end fit securely into the Luer Lok syringe hub of the rat still in the stereotaxic head holder. They were subjected to fluid-percussion TBI (266–320 mV oscilloscope (Tektronix TDS 1002 (60MHz, 2-channel digital real-time) with Trauma Inducer Pressure Transducer Amplifier) readings, 1.81–2.17 atm range calculated, consistently held at 15.5 cm pendulum height, and pressure pulse length set at 25 ms) immediately after the return of a withdrawal reflex to paw pinch. Prior to FPI induction, the device and connected tubing were filled with sterile water and checked that they were free of air bubbles. The device was prepared for the injury by delivering approximately three test pulses (confirmed by a smooth waveform on the oscilloscope) while the Luer Lok syringe hub at the end of the tubing was in the closed position. After TBI or sham injury, rats were disconnected from the fluid percussion device, and righting reflex was assessed until a normal righting reflex was observed three times (and the time at the third righting was recorded). Rats were then placed on 2% isoflurane, while wound sites were infused with bupivicaine and skin was closed with wound clips. The animals received approximately 100 mg/kg acetaminophen suppository before emerging from anesthesia. Isoflurane was discontinued, and the rats were extubated and allowed to recover in a warm, humidified incubator. When each rat was fully recovered, it was returned to its home cage with *ad libitum* food and water. All animals were monitored for signs of infection, severe neurological injury, or discomfort. Signs of discomfort or pain in rodents include persistent dormouse position and unwillingness to move, refusal to eat or drink, vocalizations when handled, posturing, aggressiveness, and polyphagia of bedding. Rats exhibiting these symptoms are humanely euthanized immediately (4% isoflurane in an anesthetic chamber followed by decapitation) to prevent pain and distress.

### Tissue collection

At the appropriate timepoint after FPI (24 h, 2 weeks, 3, 6 or 12 months post FPI), animals were anesthetized with 4% isoflurane in an anesthetic chamber and decapitated. The brain was quickly and carefully removed using bone rongeurs, and the brain regions were dissected out and either flash-frozen on dry ice (for proteins) or put into a microcentrifuge tube filled with RNALater (for gene expression). The rest of the brain was flash-frozen on dry ice and kept at −80 C.

### Working-memory Morris water maze

We used a working-memory Morris water maze (MWM) paradigm that showed learning and memory deficits persisting up to a year following the FPI model used in this study (Sell et al., 2017). On post-injury days 11–15, rats underwent MWM testing, which is described in more detail in the study by Sell et al. (2017). Briefly, the water maze consisted of a 1.8-m-diameter tank filled with water to a height of 28 cm. This height is 2 cm higher than the invisible platform, which is 10 cm in diameter and 26 cm in height. Rats received four pairs of trials for five consecutive days. Rats were assigned four starting points (N, S, E, or W) and four platform locations (1, 2, 3, or 4) in a balanced order to avoid starting points too close to the platforms. For Trial 1, rats were placed in the tank facing the wall at the assigned location and allowed 120 s to find the platform. For Trial 2, rats were immediately placed back in the same starting position and again allowed 120 s to find the platform. After Trial 2, the rats rested for 4 min in a heated enclosure, followed by the second pair of trials (using a new entry point and new platform location) and repeated until four pairs of trials were completed. All rats experienced the same sequence of start points and platform locations, which were randomly selected at the beginning of the experiment.

### RNA isolation, quality, and quantity evaluation

The quality and quantity of the total RNA sample were assessed using an Agilent Bioanalyzer with the RNA6000 Nano Lab Chip (Agilent Technologies; Santa Clara, CA).

### Gene expression microarrays

Labeled cRNA was prepared by linear amplification of the Poly(A) + RNA population within the total RNA sample. Briefly, total RNA was reverse-transcribed after priming with a DNA oligonucleotide containing the T7 RNA polymerase promoter 5’ to a d(T)24 sequence. After second-strand cDNA synthesis and purification of double-stranded cDNA, *in vitro* transcription was performed using T7 RNA polymerase. The quantity and quality of the cRNA were assayed by spectrophotometry and on the Agilent Bioanalyzer as indicated for total RNA analysis. Purified cRNA was fragmented to a uniform size and applied to Agilent Rat Gene Expression Microarray 8 × 60 (Agilent Technologies, design ID 028279 and 074036) in hybridization buffer. Arrays were hybridized at 37°C for 18 h in a rotating incubator, washed, and scanned using a G2565 Microarray Scanner (Agilent Technologies). All arrays were performed by GenUs BioSystems, Inc.

All arrays were processed using Agilent Feature Extraction software, and data were analyzed using GeneSpring GX software (Agilent Technologies) by GenUs BioSystems, Inc. To compare individual expression values across arrays, raw intensity data from each probe were normalized to the 75th percentile intensity of its array. Probes with values greater than background intensity in three or more replicates of at least one condition were filtered for further analysis. After this quality filtering, differentially expressed probes were identified with unadjusted Welch t-test *p*-values < 0.05 and fold change >1.5 fold. This combination of quality filtering, fold change filtering, and a moderate *p*-value is in accordance with the US FDA-sponsored MAQC study conclusion that there is better reproducibility when genes are ranked on fold change with a non-stringent *p*-value cutoff (Shi et al., 2006).

### Identification of eigengenes and behavioral principal component analyses

A combined dataset for probe expression data from the microarray analyses was used for all animal subjects across two brain regions (cortex and hippocampus), three injury conditions (naïve, sham, and TBI), and multiple timepoints ranging from 24 h up to 1 year post injury/surgery (n = 4 per group, N = 96 total). Data were initially curated based on problematic genes whose expression results were outside the acceptable range (flagged as “A” for absent) in either the hippocampus, the cortex, or both regions. From the 45,598 probes in the original array, 23,480 probes were excluded from the analysis based on the exclusion criteria, leaving 22,118 probes remaining for the identification of eigengenes that could be used for more robust hypothesis testing on the three-way interaction between brain region, injury condition, and timepoint.

Non-linear principal component analysis (NL-PCA) was performed using SPSS v24. Due to the large size of this dataset, where the analysis needed to be run across more than 20,000 variables, customized SPSS syntax was written to suppress graphical outputs and generate data tables with the PC loadings and percent variance accounted for. Each probe was categorized as a numeric variable with multiplying discretization and listwise deletion for any missing data. However, the dataset used was complete, and therefore no deletion was necessary to account for missing data. An initial NL-PCA was performed using 20 dimensions for factor determination, and the eigenvalues were used to determine how many dimensions to force into the model (e.g., when the eigenvalues are greater than 1.0). A total of 10 factors were assigned in the NL-PCA model. Once the eigengenes were calculated, a scree plot of the eigenvalues/variance accounted for was plotted for each PC, where factors to include were determined based on the asymptote in the scree plot (Supplementary Figure S1).

The identity of each new eigengene was determined by rank sorting the PC loadings for each probe. Individual probes were assigned only to an eigengene where their loading was the highest across all PCs. Only probes with a loading greater than |0.5| were included. Each list of probes that met the criteria for the highest loading across all PCs and was greater than |0.5| was uploaded for each eigengene individually into Ingenuity Pathways Analysis (IPA, QIAGEN, version 01-12 (01-12)). Analyses were run using the core analysis option, using the ingenuity knowledge base for probe IDs, and PC loadings as the expression outcome to identify the top canonical pathways and upstream regulators.

Second-order “meta-PCA” is a PCA that uses the PC scores from the first round of analyses to further understand the relationship between components. The meta-PCA is set up and run like a normal PCA, with the only difference being that instead of raw variables (such as gene expression or behavioral tasks) as the input, it uses components that have been identified in the previous set of PCAs, represented by PC scores of those components. This allows for disparate, multimodal data to be combined and patterns of variance explained between these components to be explored. Principal component analyses for behavioral data, as well as the second-order PCA run on PC scores from genomic and behavioral PCAs, were run using the syndRomics package in R (Torres-Espín et al., 2021).

### Hypothesis testing of PC scores

PC scores were created for each eigengene for each animal with the probe dataset (N = 96 total, n = 4 per group). Due to differences in timepoints of the samples collected from the cortex (24 h, 2 weeks, 3 months, 6 months, and 1 year) and hippocampus (2 weeks, 6 months, and 1 year), full analyses across all timepoints for hypothesis testing was performed separately for each brain region to test a two-way interaction between these three independent variables. Hypothesis testing for injury condition, timepoint, and brain region used two-way or three-way analysis of variance, as appropriate. All statistical testing on PC scores was performed in R.

## Results

### Principal component analysis: Genomics

**FIGURE 1:**
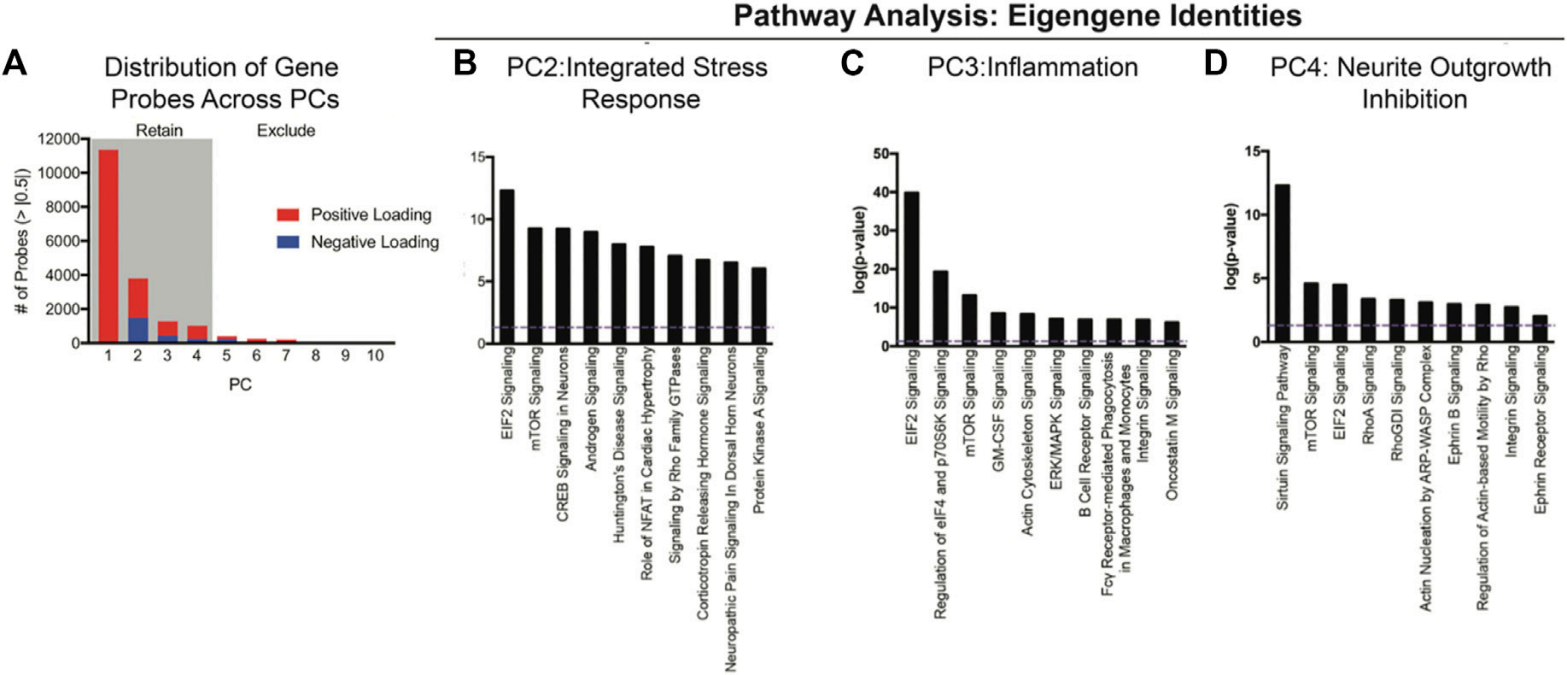
NL-PCA was performed on 22, 119 gene expression probes to reduce the complexity into eigengenes for the discovery of clusters of genes that covary together independently from one another. **(A)** First, four PCs accounted for a total of 67% of the variance in the dataset. Total probes with significant loadings greater than |0.5| were plotted based on positive (red) or negative (blue) loadings across PC1–4 = 17,438 probes. (**B–D)** Eigengenes were further analyzed using Ingenuity Pathway Analysis (IPA) to identify the gene families and cell signaling pathways that each eigengene represents. **(B)** PC2 was characterized by EIF2 and mTOR signaling, indicating an integrated stress response component. **(C)** PC3 included GM-CSF and p70S6K, indicating an inflammatory component. **(D)** PC4 included Rho pathways, indicating neurite outgrowth inhibition. Error bars = SEM.

Non-linear principal component analysis performed on 22,118 genes on 96 animals revealed four eigengenes (principal components, Figure 1). These four eigengenes accounted for 67**%** of the variance in the dataset. Using an absolute loading threshold of 0.5, we found that the first eigengene was comprised of ∼11,354 high-loading genes, with eigengenes 2, 3, and 4 represented by ∼3,806, 1,278, and 1,000, respectively. Ingenuity Pathway Analysis was then used to determine the gene families and cell signaling pathways that represented the ‘identity’ of each eigengene. This pathway analysis unveiled three interpretable eigengene components (PC2, PC3, and PC4) characterized by *integrated stress response* (PC2; EIF2-mTOR, corticotropin signaling, etc.), *inflammatory factors* (PC3; GM-CSF and PI3K-p70S6K signaling), and *neurite growth inhibition* (PC4; Rho pathways).

**FIGURE 2:**
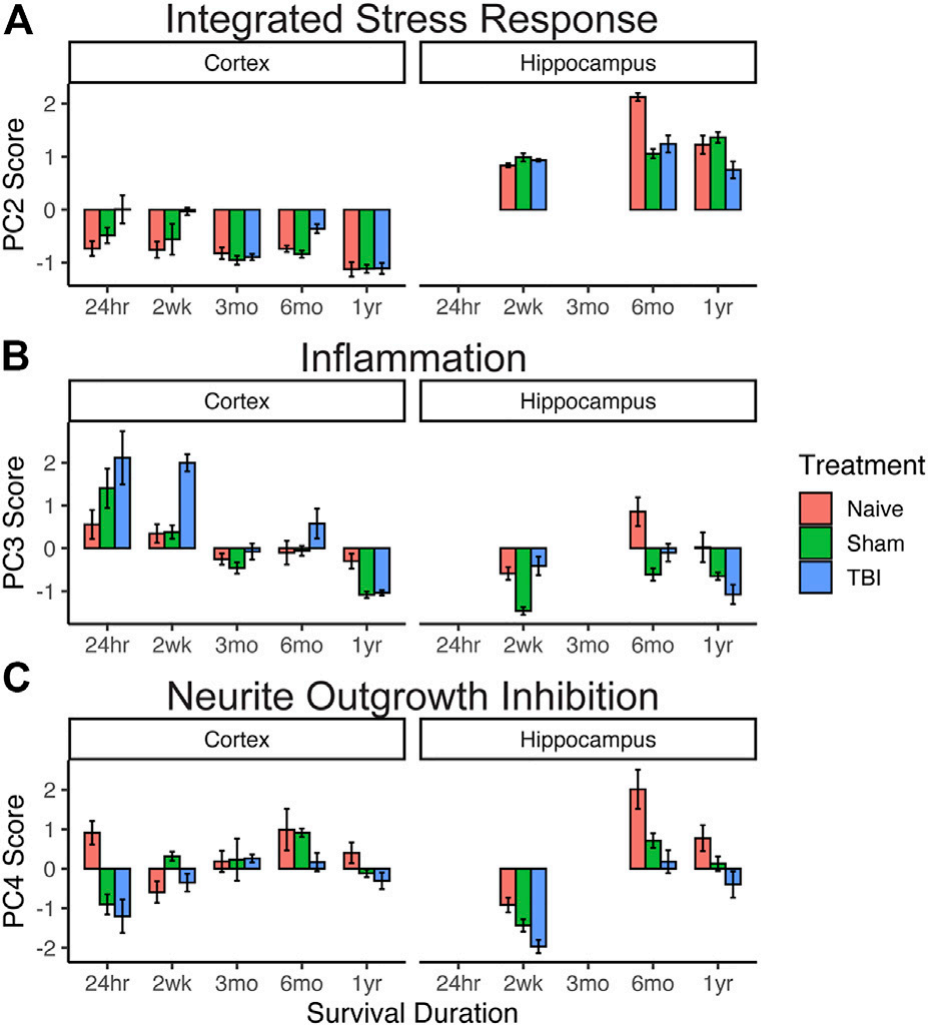
Effect of injury on genomic pathway components. Extraction of eigengene expression profiles for each animal allowed for targeted hypothesis testing for the three-way interaction between brain region x injury condition x timepoint using PC scores (n = 4 per group). **(A)** Integrated stress response. Early (24 h–2 weeks) increase in stress response gene expression in the cortex in response to TBI resolves between 3 and 12 months. **(B)** Inflammation. Inflammatory gene expression in the cortex was high in TBI subjects in early timepoints (24 h–2 weeks) but resolved by 3 months. Compared to the cortex, inflammatory gene expression in the hippocampus was reduced, with TBI patients showing significantly lower expression at 1 year than sham or naïve subjects. **(C)** Neurite outgrowth inhibition. TBI subjects showed a significant decrease compared to sham and naïve subjects in both brain regions at 6 months. Error bars = SEM.

We then used individual PC scores for each subject on PCs 2, 3, and 4 for hypothesis testing of differential eigengene expression across timepoints, injury condition, and brain region (Figure 2 **)**. For integrated stress response gene expression component (PC2), three-way analysis of variance revealed the main effects of timepoint after injury (F4,72 = 61.5, *p* < 0.001, partial η^2^ = 0.77) and brain region (F1,72 = 951.3, *p* < 0.001, partial η^2^ = 0.93). All two-way and three-way interactions between injury condition, timepoint, and brain region were also significant (Fs > 2.65, *p* < 0.05, partial η^2^ > 0.13). There was no overall main effect of injury condition (F2,72 = 2.12, *p* = 0.13, partial η^2^ = 0.06). These findings suggest an early (24 h–2 weeks) increase in stress response gene expression in the cortex in response to TBI that is resolved between 3 and 12 months post injury. Stress response gene expression is greater in the hippocampus overall than in the cortex, and by 12 months this response is significantly decreased in TBI subjects compared to sham and naïve subjects.

For the inflammatory principal component (PC3), three-way analysis of variance revealed main effects of injury condition (F2,72 = 10.2, *p* < 0.001, partial η^2^ = 0.06), timepoint after injury (F4,72 = 33.0, *p* < 0.001), and brain region (F1,72 = 19.0, *p* < 0.001). All other two-way interactions were also significant (Fs > 6.36, *p* < 0.001). Inflammatory gene expression in the cortex was high in TBI subjects in early timepoints (24 hr–2 weeks) but resolved by 3 months. Compared to that of the cortex, inflammatory gene expression in the hippocampus was reduced, with TBI patients showing significantly lower expression at 1 year than sham or naïve subjects.

For the neurite outgrowth inhibition principal component (PC4), three-way analysis of variance revealed main effects of injury condition (F2, 72 = 20.4, *p* < 0.001) and timepoint after injury (F4, 72 = 26.5, *p* < 0.001), but no main effect of the brain region (F1,72 = 3.6, *p* = 0.06). All two-way interactions were significant (Fs > 3.4, *p* < 0.01). While neurite growth inhibition generally increases over time in both brain regions and injury conditions, TBI subjects tended to have lower expression than sham and naïve subjects, and this expression significantly decreased compared to that of sham and naïve subjects in both brain regions at 6 months.

### Principal component analysis: Behavior

A separate principal component analysis was then performed on behavioral data (24 total behavioral variables). As with the genomic PCA, loadings were thresholded at the absolute value of 0.5 to determine those variables that contributed the most to the variance explained within each principal component and to aid in determining the identity of each PC. This unveiled three interpretable behavioral components characterized by broad Morris water maze measures (PC1), neuroscore and beam walk measures (PC2), and specific Morris water maze platform entry/time measures (PC3). We identified three principal components, accounting for 49.6%, 12%, and 10% of the variance, respectively. PC1 uncovered a pattern of Morris water maze variables loading together in the same direction, indicating that the most variance is explained by converging measures of working memory. PC2 consisted of neuroscore/beam balance scores loading together, and PC3 was a more specific subset of Morris water maze measures of platform performance.

**FIGURE 3:**
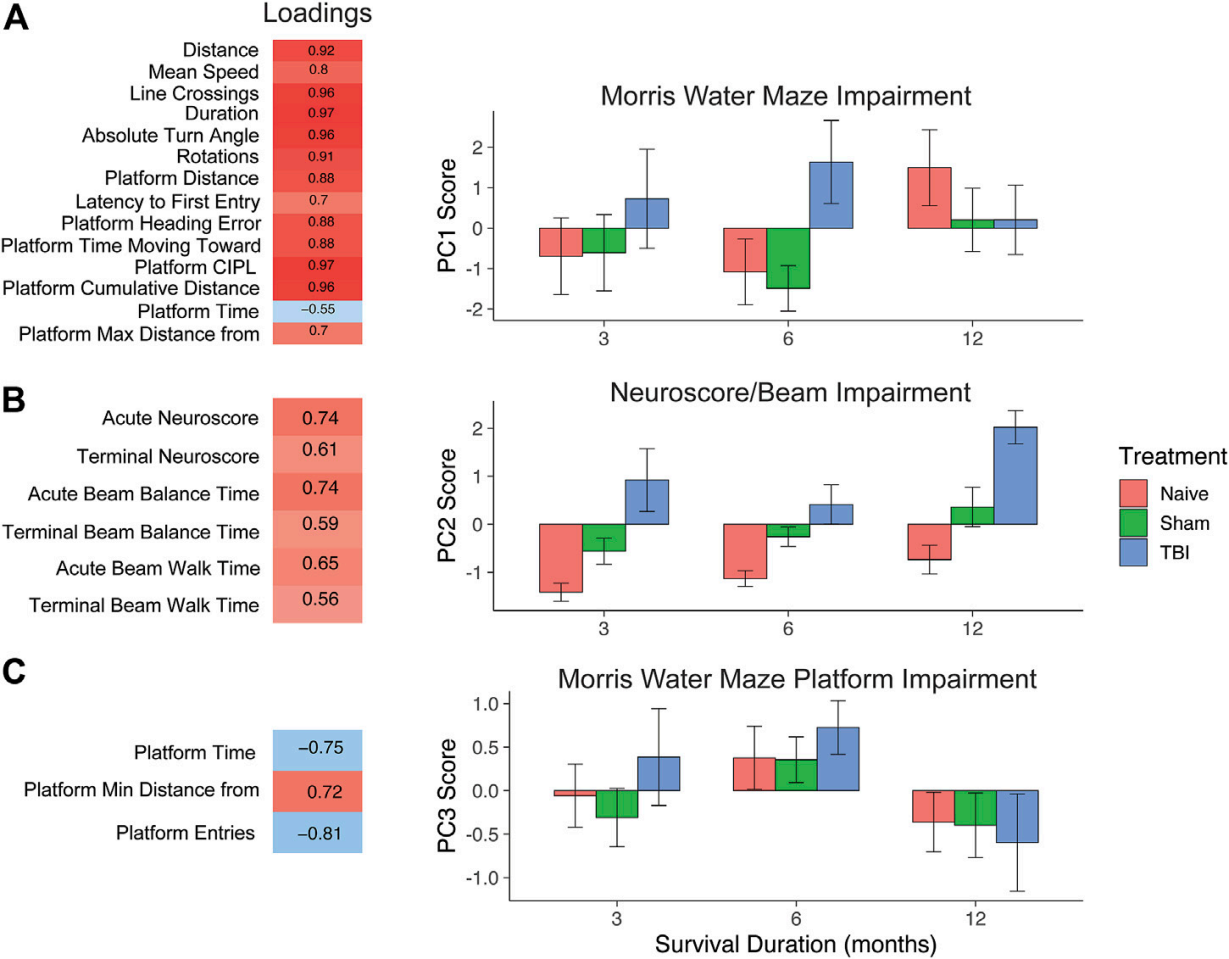
PCA on behavioral outcome measures and testing effect of injury and time with PC scores. **(A)** PC1, Morris water maze impairment. Behavioral measures that load highly (>|0.5|) reflect Morris water maze measures. Higher values indicate worse impairment. TBI induced a significant increase over sham and naïve animals by 6 months post-TBI. **(B)** PC2, neuroscore/beam impairment. High-loading variables in PC2 reflect impairment in neuroscore and beam measures. TBI significantly increased on this measure of impairment at 3, 6, and 12 months post-TBI. **(C)** PC3, Morris water maze platform impairment. High-loading variables indicate specific impairment in platform measures. There was a significant effect of timepoint, but no effect of injury, as all injury conditions followed a similar course over time. Error bars = SEM.

We then used individual PC scores for each subject to test for differences in behavioral outcome across injury condition and timepoint after injury (Figure 3). For the broad Morris water maze component (behavioral PC1), higher PC scores indicated worse outcome. Despite a robust increase in the PC1 score for TBI subjects at 6 months, there was no overall main effect of injury condition (F2, 106 = 2.0, *p* = 0.14, partial η^2^ = 0.04), no main effect of timepoint (F2,106 = 0.72, *p* = 0.49, partial η^2^ = 0.01), and no injury by the timepoint interaction (F4,106 = 1.5, *p* = 0.22, partial η^2^ = 0.03). In the neuroscore/beam balance component (behavioral PC2), higher PC scores were also indicative of worse outcome, and two-way ANOVA revealed main effects of injury condition (F2,106 = 34.1, *p* < 0.001, partial η^2^ = 0.38) and timepoint (F2,106 = 6.9, *p* < 0.01, partial η^2^ = 0.10). At each timepoint, there was a significant difference between each injury condition, illustrating a persistent impairment for TBI subjects out to 12 months. For the specific Morris water maze platform component (behavioral PC3), higher values reflected less time in the platform area and fewer entries into the platform area, and thus higher PC3 scores indicated worse performance. Similar to the broad Morris water maze measures in PC1, there was an increase in PC3 scores for TBI subjects between 3 and 6 months that resolved by 12 months. In this case though, naïve and sham subjects followed this pattern closely. Thus, there was no main effect of injury condition (F2, 106 = 0.2, *p* > 0.05, partial η^2^ = 0.01), but there was a significant main effect of timepoint (F2,106 = 4.1, *p* < 0.05, partial η^2^ = 0.07).

### Second-order principal component analysis: Integrating genomic and behavior data

**FIGURE 4:**
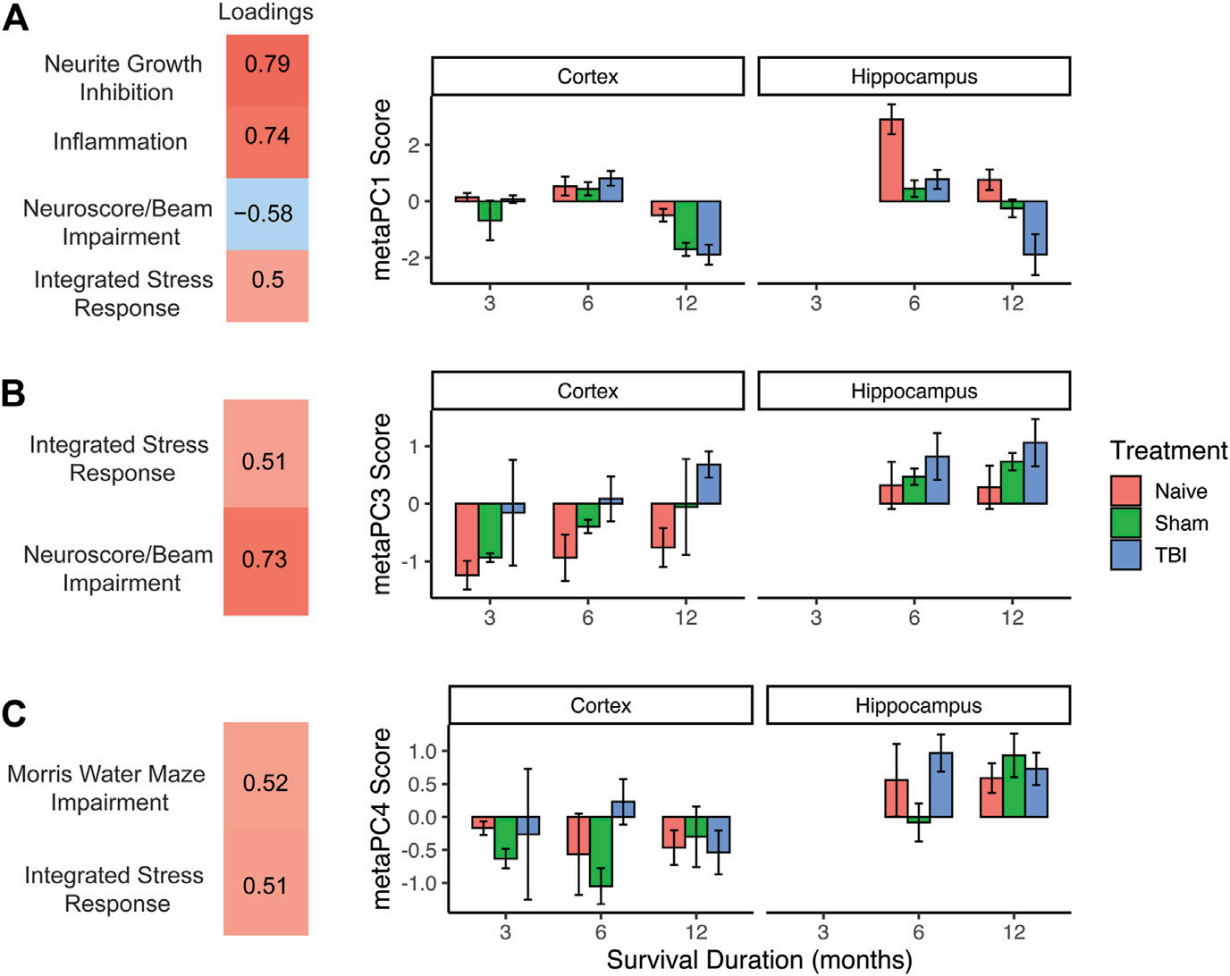
Second-order “meta-PCA” on genomic and behavioral PC scores. **(A)** meta-PC1, neuroscore/beam impairment loaded in the opposite direction as genomic components, possibly reflecting a protective effect of stress and inflammatory response. This effect is prominent in naïve animals at 6 months and 12 months, with TBI causing a robust decrease at 12 months. **(B)** meta-PC3, integrated stress response loading with neuroscore/beam impairment, indicating a stress-associated behavioral deficit. Effect of TBI was seen at alternating timepoints and was more pronounced in the hippocampus than in the cortex. **(C)** meta-PC4, integrated stress response loading with Morris water maze impairment. Higher values across all injury conditions were seen in the hippocampus, suggesting a stress-mediated effect on memory function. Error bars = SEM.

In the prior analyses, the high dimensionality of both genomic and behavioral data was reduced to three PC scores each per subject, representing latent constructs within the data. Given that PC scores are centered and scaled (similar to a z-score), this allows for the combination of these distinct domains to reveal more complex patterns in the data. The three genomic PC scores and three behavioral PC scores were used to create a second-order principal component analysis or “meta-PCA”. The meta-PCA revealed three new, interpretable meta-PCs (meta-PC1, meta-PC3, and meta-PC4) that integrated behavioral and genomic PC identities. As with previous PCAs, loadings were thresholded at an absolute value of 0.5 to aid in identifying underlying constructs. PC2 was excluded because only the strong loading variables were both behavioral PCs and thus did not provide new information about the interaction between genomic and behavioral data. These three PCs accounted for 32.7%, 16.5%, and 13.9% of the variance, respectively (Figure 4).

Meta-PC1 reflected an increase in all three genomic domains (stress response, inflammation, and neurite growth inhibition), along with a decrease in the neuroscore/beam walk impairment domain. This component suggests a possible protective effect of stress/inflammation on behavioral function. Three-way ANOVA of this meta-PC revealed main effects of injury condition (F2,44 = 14.9, *p* < 0.001, partial η^2^ = 0.40), timepoint (F2,44) = 36.2, *p* < 0.001, partial η^2^ = 0.62), and brain region (F1,44 = 14.1, *p* < 0.001, partial η^2^ = 0.24). There were also significant injury X timepoint and injury X brain region interactions (Fs > 2.8, *p* < 0.05, partial η^2^ > 0.19). There was a robust drop in this gene upregulation/behavioral improvement axis in the hippocampus for sham and TBI subjects compared to naïve subjects. For TBI subjects, there was a decrease in this component between 6 and 12 months post-TBI in both the cortex and hippocampus.

Meta-PC3 was comprised of the genomic stress response and neuroscore/beam walk impairment moving in the same direction, suggesting that this component reflects a stress-induced reduction in neuromotor function. Three-way ANOVA of this meta-PC revealed main effects of injury condition (F2,44 = 7.6, *p* < 0.01, partial η^2^ = 0.26), timepoint (F2,44) = 7.3, *p* < 0.01, partial η^2^ = 0.25), and brain region (F1,44 = 13.3, *p* < 0.001, partial η^2^ = 0.23). No interactions were significant. Across all timepoints, and in both brain regions, there was stratification by injury condition from naïve subjects with the lowest values to TBI subjects with the highest values. Regardless of timepoint or injury condition, there were higher values on this component in the hippocampus than in the cortex.

The final meta-PC consisted of the genomic stress response moving in the same direction as impairment in the broad Morris water maze component, suggesting a stress-induced reduction in working memory. Three-way ANOVA of this meta-PC revealed only a main effect of the brain region (F4,44 = 22.9, *p* < 0.001, partial η^2^ = 0.14). As seen in the pattern of expression by the brain region in the prior two meta-PCs, there were higher values on this component in the hippocampus than in the cortex, regardless of injury condition or timepoint.

## Discussion

In this study, we used multivariate analytical tools to reduce the high dimensionality of genomic and behavioral data and then integrated these multimodal data to discover unique patterns of impairment and recovery after TBI. Using PCA and pathway analysis, we found three interpretable genomic components characterized by integrated stress response, inflammatory factors, and neurite growth inhibition. Similarly, there were three behavioral components that arose, representing broad impairment in Morris water maze, impairment in neuroscore/beam balance, and more specific impairments related to Morris water maze platform entries and time in the platform area. Then, to explore how these components were interrelated, we ran a second-order PCA using the genomic and behavioral PC scores. This allowed us to further reduce the multimodal complexity to components that identified latent bio-behavioral patterns which we could compare across time after injury, injury condition, and brain region.

The first “meta-PC” revealed a negative correlation between the genomic components and neuroscore impairment, suggesting that in subjects with greater neuroscore impairment, there was an associated decrease in genes related to inflammatory signaling, stress response, and neurite growth inhibition. This finding is consistent with the notion that inflammation and/or stress response can provide neuroprotection after CNS injury (Corps et al., 2015; Russo and McGavern, 2016). One of the signaling pathways that characterized the integrated stress response identified in genomic PC2 was the androgen signaling pathway. Androgen signaling is altered by TBI, leading to mitochondrial impairment, and a recent work has suggested that pharmacological activation of the androgen signaling pathway after TBI may increase neuron survival (Crespo-Castrillo et al., 2018) and decrease lesion volume (Gölz et al., 2019). Similarly, one of the signaling pathways that were found to be a significant contributor to genomic inflammatory PC3 was granulocyte–macrophage colony-stimulating factor (GM-CSF). Despite being a pro-inflammatory cytokine, GM-CSF has been shown to induce wound healing and reduced lesion volume after TBI (Shultz et al., 2014), attenuated neurodegeneration in a model of Parkinson’s disease (Kosloski et al., 2013), and has been recently shown to improve spatial memory in a model of Alzheimer’s disease (Potter et al., 2021). Thus, meta-PC1 may reflect the neuroprotective roles of stress and inflammation after TBI.

Meta-PCs 2 and 3 were characterized by increased integrated stress response signaling positively correlating with impairment in recovery of neuromotor and memory after TBI. Genomic PC2 was characterized as being related to integrated stress response, given the robust presence of the EIF2 signaling pathway. TBI induces sustained phosphorylation of EIF2, inhibiting protein synthesis and undermining recovery of cognitive function (Hinnebusch et al., 2016; Chou et al., 2017). Our finding that genomic PC2 scores were much higher in the hippocampus is consistent with prior findings showing a specific increase in EIF2 phosphorylation in the hippocampus after TBI (Dash et al., 2015; Hood et al., 2018). Interestingly, this increased hippocampal-integrated stress response component was correlated not only with memory impairment (meta-PC3), as would be expected with hippocampal damage, but also with impairment in neuromotor recovery (meta-PC2). These findings indicate that targeting stress response signaling, such as EIF2, may have a broad beneficial effect on recovery after TBI, as other studies have previously shown (Dash et al., 2015; Chou et al., 2017).

One goal of this study was to integrate separate data modalities to increase our sensitivity to possible effects of injury condition and timepoint. We and other researchers have shown that when analyzing a large number of outcome measures (as is often the case in TBI research), there is a high risk of selection bias for reporting only the handful of univariate measures that may be significant (Haefeli et al., 2017). Instead, using multivariate tools such as PCA allows us to model the variance between many outcomes together and helps to ensure that the patterns, effects, and interactions that we detect are robust and stable. By avoiding the possible pitfalls of spurious univariate effects, we can also aim to generate results that are more likely to be reproducible. Indeed, this workflow was able to produce results that are consistent with prior work and thus lends validity to this multivariate, multimodal approach. Future work that integrates genomic, behavioral, and/or proteomic data in this way may prove useful in identifying novel and previously undiscovered patterns at the intersection of these bio-behavioral axes. PC scores often being in opposite directions between the hippocampus and cortex offers one such area of interest; further work will be necessary to uncover the potential opposing roles for these brain regions when viewing them through a multivariate lens, in which genomic pathways and behavioral recovery over time after TBI are considered in an integrated, multidimensional model.

Large-scale data efforts such as this one also necessitate that the data be well-organized and accessible. To this end, we used the Open Data Commons for Traumatic Brain Injury (ODC-TBI, https://odc-tbi.org (Chou et al., 2021)) as a data repository and data management tool. The ODC-TBI allows members to host and organize their data in a private online space, and then when data are used in a publication, one can publish the dataset itself as a companion to the article. A unique data object identifier (DOI) is minted for the dataset, so that as others reuse the data in future work, the data can be cited and the creators can get credit for their work. As the NIH has mandated that by 2023 all datasets underlying NIH-funded work be made public, tools such as the ODC-TBI will be crucial in implementing these new policies. As such, the dataset from the current study will be made public and can be found at https://odc-tbi.org.

Overall, this study demonstrates a practical analytical workflow for integrating and interpreting multimodal data and illustrates how these different forms of data can be combined to produce new insights. While behavior and genomic results were shown here, this large-scale effort from the Moody Project Team also generated proteomics data. Future studies may also be able to include these data to reveal further multidimensional patterns of impairment and recovery after TBI.

## Data Availability

The data presented in the study are deposited in the Open Data Commons for TBI repository (odc-tbi.org), under the DOI 10.34945/F5HG6B.
