# Supplementary figures and images for: Data-driven approach to integrating genomic and behavioral preclinical traumatic brain injury research
Source: Front Bioeng Biotechnol. 2023 Jan 10;10:887898. doi: 10.3389/fbioe.2022.887898 (PMC9871446; doi:10.3389/fbioe.2022.887898)

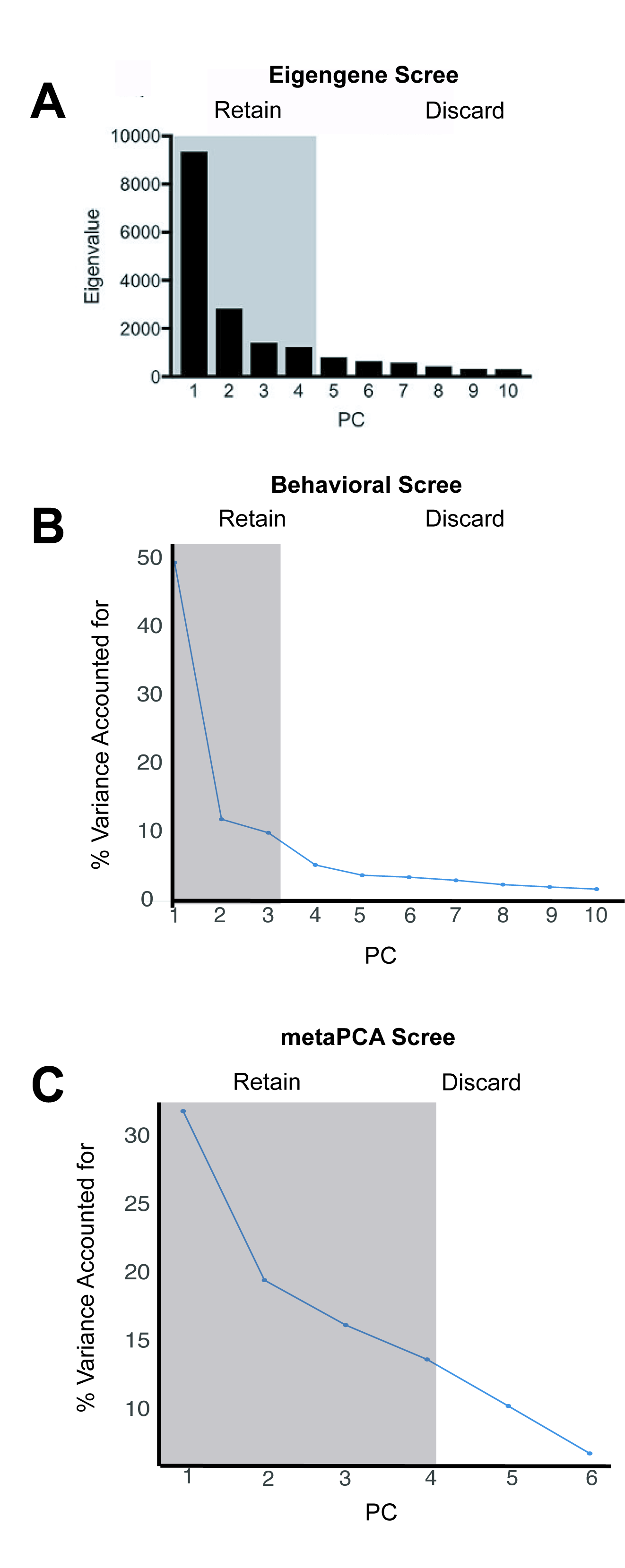

Supplement: Supplementary file 1 [file Image1.tif]
